# Effect of the menstrual cycle on circulation during combined spinal-epidural anaesthesia

**DOI:** 10.1186/s12871-018-0573-x

**Published:** 2018-08-17

**Authors:** Hua Lin, Wen-zhi Li

**Affiliations:** 1 Department of Anesthesia, Tianjin Medical University General Hospital Airport Hospital, Tianjin, 300300 China; 2 Department of Anesthesia, The Second Affiliated Hospital of Harbin Medical University, Harbin, 150000 Heilongjiang China

**Keywords:** Female hormone, Luteal phase, Follicular phase, Circulatory system

## Abstract

**Background:**

From adolescence to menopause, hormone levels during the menstrual cycle affect various body systems, from the cardiovascular system to the water and electrolyte balance. This study investigated the effect of different phases of the menstrual cycle on circulatory function relative to changes in body position and combined spinal-epidural anaesthesia (CSEA).

**Methods:**

Forty-six women were selected who underwent scheduled gynaecological surgery, were classified as American Society of Anesthesiology (ASA) I-II, and met the test criteria. The sample was divided into the follicular and corpus luteal groups. Preoperative heart rate and blood pressure measurements were taken from the supine and standing positions. Heart rate measurements as well as systolic, diastolic, and mean blood pressure measurements were taken upon entering the operating room, at the beginning of the spinal-epidural anaesthesia, and 10, 20, and 30 min after anaesthesia was administered.

**Results:**

The heart rates of patients in the corpus luteal group were higher than those of patients in the follicular group both before and after anaesthesia (*P* <  0.05). Significantly more ephedrine was used during the first 30 min of CSEA in the corpus luteal group than in the follicular group (*P* <  0.05).

**Conclusions:**

Although the effect was slight, women in the follicular phase were better able to compensate and tolerate circulatory fluctuations than those in the luteal phase.

## Background

From adolescence to menopause, hormone levels during the menstrual cycle affect various body systems, from the cardiovascular system to the water and electrolyte balance. The periodically fluctuating hormone levels affect the endometrium, causing the menstrual cycle to be divided into three phases: menstruation, the follicular and luteal phases. Previous studies have primarily focused on the long-term effects of oestrogen and progesterone on the cardiovascular system, including the reduction in atherosclerosis, coronary heart disease and the pathogenesis and progression of hypertension. Recent studies have found that the heart rate is higher during the luteal phase than during the follicular phase, while the heart rate varies less during the luteal phase than during the follicular phase. Most of these studies focused on how hormone levels affect the body during states of rest, stress, or exercise [1–9]; however, no report has addressed the effect of hormone levels on the circulation during body position changes or during anaesthesia. In this study, we investigated how body position changes and combined spinal-epidural anaesthesia (CSEA) affect circulatory function during the stages of the menstrual cycle.

## Methods

### Study participants

Fifty women aged between 25 to 45 years, who underwent a scheduled gynaecological myomectomy between March and August 2015, and were classified as American Society of Anesthesiology (ASA) I-II were initially selected. Four of the women rescheduled their surgery; therefore, 23 were assigned to the follicular group (6–12 d after their last menstrual period), and 23 were assigned to the corpus luteal group (16–24 d after their last menstrual period). All patients met the inclusion criteria. Of these patients, six required general anaesthesia; therefore, the final totals included 21 patients in the follicular group and 19 patients in the corpus luteal group. All patients’ menstrual cycles ranged from 28 to 32 d, and none had used any cardiovascular or hormonal drugs for ≥6 months. Patients with histories of smoking, mental illness, puncture site infection, peripheral nervous system disease, diabetes, spinal deformity, or allergies to local anaesthesia were excluded. The ethics committee of our hospital approved this study, and all patients signed an informed consent form.

### Anaesthesia methods and indicators

The same group of gynaecologists operated on all patients, and the same anaesthesiologist provided the spinal-epidural anaesthesia. Patient age, height, body mass, body mass index (BMI), and menstrual cycle were recorded. All patients fasted from food and water for 8 h before the procedure, and their blood pressures were measured from the supine and standing positions and recorded on the morning of the surgery (7:00–10:00 am). For this, patients assumed the supine position in a quiet room for 10 min before having their systolic and diastolic blood pressures, mean pressures, and heart rates (T_0_) measured. The patients then stood, and 30 s later, their systolic, diastolic, and mean blood pressures and heart rates (T_1_) were measured. This was repeated twice to calculate an average. Patients were not infused before the operation without special circumstances. After entering the operating room, the patient assumed the supine position for 5 min. Venous access was initiated, and Ringer’s lactate solution was intravenously infused at 500 ml/h over the first hour. Systolic, diastolic, and mean blood pressures and heart rate were recorded upon entering the operating room (T_2_), at the beginning of anaesthesia (T_3_), and at 10 min (T_4_), 20 min (T_5_), and 30 min (T_6_) after receiving anaesthesia. Before starting anaesthesia, the patients moved from a supine position to right lateral recumbency, and L3–4 was selected as the puncture site. The median approach method was used for the CSEA puncture. After observing free flow of cerebrospinal fluid, 3 ml of 0.5% hyperbaric bupivacaine was injected. After removing the spinal anaesthetic needle, epidural catheterisation was performed. After the catheter was fixed, the patient was repositioned into the supine position, and the sensory block level was adjusted to approximately T_6_. After determining the anaesthetic effect, the operation began 30 min after the start of anaesthesia (T_6_). If the patient’s blood pressure fell below 90/60 mmHg, and their heart rate was less than 90 beats/min, 6 mg of ephedrine was injected intravenously. If their blood pressure was below 90/60 mmHg, but their heart rate was not less than 90 beats/min, 0.25 mg of metaraminol was injected intravenously. The drugs were given repeatedly if needed.

### Statistical analysis

Data were analysed using SPSS 19.0. Continuous data with a normal distribution were expressed as the means ± standard deviations. Comparisons between the groups were performed with t-test, and *P* < 0.05 was considered significant.

## Results

### General patient information

No significant differences were found in patient age, height, body mass, or BMI (*P* > 0.05). No significant differences were observed in the anaesthetic plane, the infusion volume within 30 min after anaesthesia, or the food and water fasting time (*P* > 0.05) (Table 1).

**Table 1:** General patient condition

	Age (years)	Height (cm)	Body weight (kg)	BMI (kg/m^2^)	Infusion volume (ml)	Fasting food and water time/h
Follicle group (*n* = 21)	41 ± 5.7	161 ± 6.4	60 ± 15	23 ± 4.0	289 ± 57.0	12.48 ± 0.81
Corpus luteal group (*n* = 19)	39 ± 4.1	162 ± 5.7	59 ± 14	23 ± 3.7	300 ± 45.0	11.54 ± 0.66

### Blood pressure and heart rate associated with the supine and standing positions

No significant difference was observed between the corpus luteal and follicular groups for the blood pressure and heart rate measurements taken in the supine position (T_0_). After the patients moved from the supine to standing position (T_1_), the heart rate of the corpus luteal group was significantly higher than that of the follicular group (*P* < 0.05). No significant difference in blood pressure change was observed for the standing position between the two groups (*P* > 0.05, Table 2).

**Table 2:** Blood pressure and heart rates for patients in both groups while standing

	Variable	Follicular phase	Luteal phase	*P-*value
T_0_	Heart rate (beats/min)	68 ± 8.9	73 ± 9.7	0.06
	SBP (mmHg)	114 ± 11.3	111 ± 8.3	0.27
	DBP (mmHg)	71 ± 9.4	69 ± 9.2	0.34
	MBP (mmHg)	86 ± 9.7	83 ± 7.4	0.28
T_1_	Heart rate (beats/min)	86 ± 12.5	93 ± 9.0^*^	0.04
	SBP (mmHg)	115 ± 12.5	112 ± 10.9	0.35
	DBP (mmHg)	74 ± 10.0	72 ± 8.1	0.41
	MBP (mmHg)	88 ± 10.5	86 ± 8.4	0.37

*P < 0.05 compared with the follicular group

### Blood pressure and heart rate before and after combined spinal-epidural anaesthesia

No significant differences in blood pressure or heart rate were found when patients entered the operating room (T_2_), at the beginning of anaesthesia (T_3_), at 10 min (T_4_), or at 20 min (T_5_) into the anaesthesia (*P* > 0.05). However, at 30 min into the anaesthesia (T_6_), the heart rates of the corpus luteal group were significantly higher than those of the follicular group (*P* < 0.05, Table 3).

**Table 3:** Blood pressure and heart rate before and after receiving anaesthesia by group

	Variable	Follicular phase	Luteal phase	*P*-value
T_2_	Heart rate (beats/min)	84 ± 15.1	84 ± 11.8	0.99
	SBP (mmHg)	125 ± 15.8	127 ± 13.5	0.56
	DBP (mmHg)	78 ± 14.3	79 ± 11.9	0.71
	MBP (mmHg)	93 ± 14.0	95 ± 12.0	0.64
T_3_	Heart rate (beats/min)	81 ± 14.5	84 ± 9.7	0.47
	SBP (mmHg)	124 ± 16.5	125 ± 12.2	0.77
	DBP (mmHg)	77 ± 16.7	79 ± 12.2	0.71
	MBP (mmHg)	93 ± 15.1	94 ± 11.5	0.70
T_4_	Heart rate (beats/min)	80 ± 14.9	83 ± 11.9	0.44
	SBP (mmHg)	120 ± 15.4	120 ± 16.9	0.98
	DBP (mmHg)	71 ± 14.3	71 ± 12.4	0.91
	MBP (mmHg)	87 ± 12.8	88 ± 13.0	0.94
T_5_	Heart rate (beats/min)	73 ± 11.2	76 ± 11.5	0.35
	SBP (mmHg)	110 ± 13.9	118 ± 14.2	0.09
	DBP (mmHg)	66 ± 11.9	69 ± 11.2	0.38
	MBP (mmHg)	81 ± 12.0	85 ± 12.0	0.21
T_6_	Heart rate (beats/min)	71 ± 10.8	77 ± 10.4^*^	< 0.05
	SBP (mmHg)	107 ± 8.9	112 ± 13.7	0.20
	DBP (mmHg)	63 ± 9.3	67 ± 11.6	0.15
	MBP (mmHg)	78 ± 7.9	82 ± 11.0	0.12

*P < 0.05 compared with the follicular group

### Vasoactive drug dose applied during the combined spinal-epidural anaesthesia

The amount of ephedrine used in the corpus luteal group within 30 min after starting anaesthesia was significantly greater than that used in the follicular group (*P* < 0.05). No significant difference was found in the amount of metaraminol used between the follicular and corpus luteal groups within 30 min after starting anaesthesia (*P* > 0.05). Hypotension occurred more often in the luteal group than in the follicular group (Table 4).

**Table 4:** Vasoactive drug doses used in patients within 30 min after beginning anaesthesia

Variable	Follicular phase	Luteal phase	*P*-value
Ephedrine (mg)	2.00 ± 0.63	5.68 ± 1.07^*^	0.02
Metaraminol (mg)	0.012 ± 0.012	0.026 ± 0.018	0.58
Hypotension episode(time)	8	14	/

*P < 0.05 compared with the follicular group

## Discussion

This study investigated the cardiovascular regulation and compensation abilities after preoperatively fasting from food and water, under preoperative stress conditions, and under CSEA. No significant differences were found in the various circulatory indicators between patients in the supine position; however, when the supine position was changed to the standing position, the patients’ heart rates increased, and this effect was obvious during the luteal phase. After starting anaesthesia, both groups’ heart rates decreased; however, the heart rates of patients in the luteal phase were higher than those of patients in the follicular phase. Hypotension occurred more often in the luteal phase than in the follicular phase, and the required vasoactive drug dosage increased.

Female hormones include oestrogen and progesterone, and their receptors are widely distributed across various tissues and organs. Studies have found that the oestrogen β-receptor is widely distributed and that this receptor helps to regulate biosynthetic functions in tissues and cells, blood flow in tissues and organs, and body temperature. Conversely, progesterone plays the opposite role [10]. Oestrogen affects regulating the body’s circulation via three mechanisms: by increasing the responsiveness of endothelium-dependent bradykinin-mediated vasodilation and norepinephrine-mediated vasoconstriction, by upregulating nitric oxide (NO) synthase (this mechanism’s effect is controversial), and by regulating the sympathetic nervous system activity by affecting norepinephrine synthesis and the number and sensitivity of α-adrenergic receptors. Conversely, progesterone weakens the responsiveness of endothelium-dependent vasodilation to oestrogen.

Changes in posture cause much of the blood to be redistributed, leading to circulatory fluctuations. Perfusion in important organs decreases, exciting the sympathetic nervous system and the carotid sinus aortic arch baroreceptors, thus causing the heart rate to increase and the heart to work harder in order to more effectively circulate blood to the body [10–13]. Similarly, CSEA inhibits the sympathetic nerves, dilating the peripheral blood vessels below the anaesthetic plane and reducing the amount of blood returning to the heart.

Although posture changes and CSEA cause similar physiological changes, i.e., a reduced amount of blood returning to the heart over a brief time, the body must make corresponding adjustments to adapt to circulatory system changes. However, compared with circulatory fluctuations caused by preoperative posture changes, CSEA causes the blood vessels in the lower limbs to dilate and weakens the compensatory and regulatory functions below the body’s anaesthetic plane. This relies more on the compensatory function above the anaesthetic plane and regulation via exogenous vasoactive substances to effectively circulate blood and oxygen to the vital organs and tissues. Therefore, differences in the body’s regulation during the different menstrual phases became more obvious, and the vasoactive drug dose (e.g., ephedrine) used in the corpus luteal group increased. In a previous study, when patients were under the influence of mental stress, the decrease in peripheral vascular resistance and the increase in cardiac work in the corpus luteal group supported our hypothesis [14]. The results of this study showed that regardless of body position changes or whether the measurement was taken during or after anaesthesia, patients’ heart rates during the luteal phase were higher than those of patients during the follicular phase, and this result was obvious in the standing position and at 30 min after anaesthesia. This result may have occurred because during the follicular phase, oestrogen inhibits the sympathetic nervous system; however, during the luteal phase, progesterone plays the opposite role by allowing oestrogen to increase sympathetic nervous system activity. Moreover, the first peak of the preovulatory oestrogen level was higher than the second peak of the postovulatory oestrogen level. In addition, oestrogen increases the sensitivity of the vasoconstriction response to the action of norepinephrine. Therefore, during the luteal phase, the vasoactive drug dose used in the patients within 30 min after anaesthesia was greater than that used during the follicular phase. We hypothesize that women have a stronger compensatory and regulatory capacity during the follicular phase, which might be because the oestrogen level during the follicular phase is higher than that during the luteal phase, and short-term circulatory fluctuations might be primarily due to the endogenous norepinephrine-mediated vasoconstriction response. Therefore, the patients’ heart rates were lower during the follicular phase than during the luteal phase, and the demand for vasoactive drugs during the follicular phase was lower than that during the luteal phase. Although the heart rates of patients in the luteal phase were slightly higher than those of patients in the follicular phase 30 min after anaesthesia, this difference is limited to statistical significance and is not clinically significant. The menstrual cycle is not a major factor in choosing the operative period. We hypothesised that the menstrual cycle may have more significance for women with circulatory complications in responding to position changes and anaesthetic effects.

Most studies have focused on the long-term effects of oestrogen on the circulatory system, and the reduced occurrence and progression of atherosclerosis, coronary heart disease, and hypertension. Recently, researchers have been paying more attention to the female hormones’ short-term regulating of the circulatory system. Mckinley et al. divided healthy premenopausal women into two groups: those in the follicular phase and those in the luteal phase. A 24-h dynamic electrocardiogram was measured, and patient heart rates and RR interval variability (RRV) were recorded. These authors concluded that heart rate during the follicular phase was lower than that during the luteal phase, and both low-frequency and high-frequency RRV were higher than their counterparts during the luteal phase, which strongly supports the hypothesis that female hormones affect cardiovascular regulation by regulating cardiac autonomic nerve function [11]. In a study on postural orthostatic tachycardia syndrome (POTS), patients were divided into two groups: the early follicular phase and the middle luteal phase. Heart rate, peripheral vascular resistance, stroke volume per stroke (SV), and cardiac output (CO) were measured for the supine and standing positions after 2 h. In a control group from the general population, no significant differences were observed in the various indicators of the two phases. Patients with POTS in the experimental group showed higher oestrogen levels during the middle luteal phase than those during the early follicular phase, and this result corresponded to increased plasma renin and aldosterone secretion, which led to increased CO, SV, and heart rates. Furthermore, standing endurance was increased, and syncope was decreased. These authors concluded that hormone level fluctuations during the menstrual cycle affect the renin-angiotensin-aldosterone system and haemodynamic stability [2]. The increased heart rate is consistent with our conclusion; however, the changes in blood pressure differed, possibly because of the defect in vascular regulatory function among patients with postural orthostatic tachycardia syndrome. Choi et al. divided the follicular phase of the menstrual cycle into the early and late follicular phases to compare the cardiovascular system responses during movement and resting. Both oestrogen and progesterone were at lower levels during the early follicular phase than during the late follicular phase. This study found that blood pressure, heart rate, SV, and CO during the early follicular phase at movement and rest were higher than those during the late follicular phase, while peripheral vascular resistance was higher during the late follicular phase. In addition, the late follicular phase was more sensitive to exogenous norepinephrine than the early follicular phase. In that study, the blood pressure, heart rate, and sensitivity to exogenous vasoactive drugs in the late follicular phase group, which showed higher oestrogen levels, were consistent with the experimental results of the current study [3]. Gordon et al. studied the responsiveness of the cardiovascular system during different menstrual cycle phases under the influence of mental stress. That experiment was divided into the early follicular, late follicular, and luteal phases. The cardiac index (CI) of the luteal phase was significantly increased compared with the other two groups, whereas the vascular resistance index (VRI) was significantly decreased. The oestrogen and adrenaline levels during the luteal and late follicular phases were both higher than those during the early follicular phase. Oestrogen does not directly affect the CI or VRI, but it affects adrenaline sensitivity, thereby indirectly affecting haemodynamic stability. These authors concluded that haemodynamic stability depends more on the sensitivity to menstrual cycle-related adrenaline than to the hormonal level [14, 15]. The early follicular phase is also part of the menstrual cycle and women normally do not undergo surgery during their menstrual period. Our experiment did not collect data on the early follicular phase; however, the increased sensitivity to epinephrine during the period with high hormone levels supports our experimental results. Krejza et al. studied the relationship between the regulatory function of the cerebral blood vessels and female hormone levels. They collected data on the 5th, 13th, and 26th day of the menstrual cycle and recorded the plasma oestrogen concentration and bilateral carotid artery blood flow under normal conditions and after intravenously injecting 1000 mg of acetazolamide. These authors concluded that the increased carotid artery blood flow was associated with the degree of increase in the plasma oestrogen concentration [16, 17]. In general, oestrogen increases the sensitivity of endogenous and exogenous vasoactive substances and enhances the regulatory capacity of the cardiovascular system.

This study had the following limitations. First, neither oestrogen nor progesterone levels were measured. Second, we did not conduct a study on the different menstrual cycles of the same participants. Instead, we selected patients who had scheduled gynaecological surgeries and divided them into groups based on their different menstrual cycles. Third, we did not measure the specific excitability indexes of the sympathetic and parasympathetic nervous systems or the plasma concentration of norepinephrine. Fourth, we cannot exclude the transient effect of ephedrine on heart rate. Fifth, this study on women without circulatory complications under CSEA at different menstrual cycle stages and fluctuations may have more significance for women with circulatory complications.

## Conclusion

Although the effect is slight, women may be better able to compensate and tolerate circulatory fluctuations during the follicular phase than during the luteal phase.

## Abbreviations

BMI: Body mass index
CI: Cardiac index
POTS: Postural orthostatic tachycardia syndrome
RRV: RR interval variability
VRI: Vascular resistance index
